# The Proper Definition of the Word “Cure,” as Applied to Medicine

**Published:** 1872-05

**Authors:** Paul F. Eve

**Affiliations:** Professor of Operative and Clinical Surgery in the University of Nashville


*The Proper Definition of the Word "Cure," as Applied to Medicine.* By Paul F. Eve, M.D., Professor of Operative and Clinical Surgery in the University of Nashville.

To the unprofessional, the science of medicine consists in studying names for diseases, and prescribing remedies. People come to us to find out what ails them, and to obtain what they believe will cure them. They never consider that every case is a problem to be solved for itself, requiring special examination, due deliberation, and study; and then, after all, it may embarrass the most skillful, demanding consultation to determine what is best to be done. A doctor versed in the nomenclature of his profession is always preferred to one familiar only with its therapeutics, as everybody is ready to prescribe the well-known remedies for any disease, however obscure or formidable.

A morbid condition is never considered to be a modification of the natural phenomena of life—simply a perversion of the healthy functions—but is attributed to some noxious agent in the system, a certain something superadded, which must be removed, or counteracted by appropriate medicines, before health can be restored. For instance, an inflamed finger suggests the name of whitlow, and, without inquiring into the cause producing it—alike regardless, too, of constitutional peculiarity, the age, sex, habits, season, state of health, stage of inflammatory action, or what tissue may be affected, whether the skin, the cellular tissue, sheath of the tendons, or the periosteum, etc., simply because it once did good in a case, a small fly-blister (it may be) is recommended, with the most positive assurance that, in six hours, the *felon*, the source of all the pain, may be taken out with the point of a needle or scalpel.

The less medicine a physician prescribes the more unpopular he becomes; the more he trusts to the efforts of nature, in relieving his patients, the less will he be appreciated; and he who ventures to decide, in any case, that the best thing to be done for it is nothing at all, may as well at once retire from practice. Yet is the assertion most true, the older a practitioner becomes, the less confidence has he in medicines; and, as a class, it is proverbial how little physic doctors take themselves.

In a recent discourse to his church, a minister of this city, and he holding a medical diploma, made the ungracious imputation upon us for attending calls on the Sabbath, because, said he, such patients ought not to be indulged on that day; but even he writes: "The doctor cannot always diagnosticate a *cure*." Again, he has "devoted years accumulating knowledge on the subject of *curing diseases*."

That this is a true description of medicine in the estimation of the public, no one, we think, will deny. The whole of our science, if they admit we have any at all, consists simply in finding out what ails the sick, and then trying to cure him by remedies for his disease.

In yielding to popular prejudice, our profession has been placed in a false position, for which we ourselves are not wholly blameless. Practitioners of medicine are looked upon as curers of diseases and healers of wounds; and if we fail—as assuredly we must, and ever will, in the very nature of things—we ought not to complain that efforts are occasionally made to hold us responsible for bad results in practice. It is certainly the interest of our patients to do so, and as the world is now estimated by a moneyed valuation, these suits must be expected. Counting, then, the number of cases of alleged mal-practice, the question naturally arises if the time has not come for us to take the true position, that one by which this unpleasantness can be prevented, sustained, too, as it is, by truth, the aim of all honest men, and sanctioned by the highest of all authority, viz., *that in caring for the sick we do not profess to cure them.* No minister of the gospel promises to save the souls of his hearers by his preaching, or by any other human means; nor can any surgeon produce the plasma to heal the slightest wound, no obstetrician deliver a woman, nor any physician restore a patient to health, without the *vis medicatrix naturæ*, or, what we more familiarly term, nature. If the patient have not the constitution, the power within himself, to resist morbid action, no man living can give it to him. Then why shall we assume, or encourage the prevailing opinion, that we cure—we heal? Away with all false pretensions—the arrogant assumption of functions beyond the power of man; and let us be thankful that we are even humble instruments in the great and good work of promoting health and prolonging life; ever acknowledging, as we should, that it is God alone who healeth *all* our diseases.

In this definition of the word *cure*, as applied to medicine, it is not denied but that there are therapeutic agents much better adapted to relieve certain affections, or morbid conditions of the body, than are others; or that it is wrong to search after, or to engage in preparing such medicines. The great probability, however, is, that there are no specifics. Mercury, we admit, is apt to salivate; sulphur to destroy the source of the itch; atropia to dilate the pupil; acids to neutralize alkalies, and quinine to prevent chills and fever, etc.; but as diseases, some of which are self-limited, vary according to season, latitude, condition of patient, their progress, etc., so there can never be a remedy for any one of them, and no such thing as a *cure.* The world is now about six thousand years old, and does not yet acknowledge one. The offer of thousands upon thousands of francs, by Monsieur Briant, of Paris, for a cure for cholera, has not yet been awarded, and it may safely be predicted never will. For, as every one is recognized by his own peculiar countenance, so may it reasonably be inferred that he also possesses a special organization, a constitution and system *sui generis*, distinct and different from all others. Daily experience teaches us that what is food for one man may poison another. No medicine whatever will affect any two persons precisely alike. Even the number of actions from a dose of salts, the most astute physician will not venture to predict. How, then, can it be possible, from these indisputable facts, the idiosyncracies of individuals, and variability of diseases themselves, even in epidemics, to prescribe for the mere name of an affection? Yet those who do this are legion, and their advertisements alone, independent of the sales of proprietary medicines, ought to be sufficient to support a pretty strong government.

By the definition of the word "*cure*," as applied to medicine proposed and advocated by this communication, let it not be inferred that modern practice is a do-nothing system. By no means; far different. It does not simply amuse while nature cures. Knowing how little can be done when the house is in flames, we are bestowing more attention upon hygiene and the preservation of health. If it cannot cure, it obviates the necessity for resorting to too much medicine. Its master-work is the *prevention* of diseases, by investigation into their causes. When cholera was announced, a few years ago, to have been imported into Blackwell's Island, New York harbor, the Faculty of that city proposed to extinguish it in five days. By disinfectants, cleanliness, and putting the patients into tents, etc., in three days the threatened epidemic was at an end.

We are aiming, not to cure, as people will insist we are, but rather to extinguish the spark, to check the incipiency of attacks, to cut short, to divert morbid action. *Obsta principiis* is our motto, and medical science teaches that we do abort, jugulate, arrest, and thus control, many ills that flesh is heir to. Then, by the more chary use of the word "cure," in its application to the practice of medicine, our profession will be better understood, our patients expect less from us, we will be acting more honestly, and consequently made happier. Truth should ever be our aim, and if in error, a candid confession will do us good, and it is no reflection to acknowledge that the province to cure the body, like the salvation of the soul, belongs alone to Him who made both.—*Nashville Journal.*

